# EEG Headset Evaluation for Detection of Single-Trial Movement Intention for Brain-Computer Interfaces

**DOI:** 10.3390/s20102804

**Published:** 2020-05-14

**Authors:** Mads Jochumsen, Hendrik Knoche, Troels Wesenberg Kjaer, Birthe Dinesen, Preben Kidmose

**Affiliations:** 1 Department of Health Science and Technology, Aalborg University, 9220 Aalborg, Denmark; bid@hst.aau.dk; 2 Department of Architecture, Design and Media Technology, Aalborg University, 9000 Aalborg, Denmark; hk@create.aau.dk; 3 Department of Neurology, Zealand University Hospital, Roskilde, Denmark. Department of Clinical Medicine, University of Copenhagen, 2200 Copenhagen, Denmark; twk@regionsjaelland.dk; 4 Department of Engineering—Electrical and Computer Engineering, Aarhus University, 8200 Aarhus, Denmark; pki@eng.au.dk

**Keywords:** movement intention, brain–computer interface, movement-related cortical potential, neurorehabilitation

## Abstract

Brain–computer interfaces (BCIs) can be used in neurorehabilitation; however, the literature about transferring the technology to rehabilitation clinics is limited. A key component of a BCI is the headset, for which several options are available. The aim of this study was to test four commercially available headsets’ ability to record and classify movement intentions (movement-related cortical potentials—MRCPs). Twelve healthy participants performed 100 movements, while continuous EEG was recorded from the headsets on two different days to establish the reliability of the measures: classification accuracies of single-trials, number of rejected epochs, and signal-to-noise ratio. MRCPs could be recorded with the headsets covering the motor cortex, and they obtained the best classification accuracies (73%−77%). The reliability was moderate to good for the best headset (a gel-based headset covering the motor cortex). The results demonstrate that, among the evaluated headsets, reliable recordings of MRCPs require channels located close to the motor cortex and potentially a gel-based headset.

## 1. Introduction

Brain–computer interfaces (BCIs) have been proposed as a means for control of assistive devices and communication for patients with severe disabilities, such as spinal cord injury and amyotrophic lateral sclerosis (ALS) [1,2,3]. More recently, BCIs have been investigated for motor rehabilitation of patients with neural injuries such as stroke or spinal cord injury [4,5,6,7,8]. It is possible to induce neural plasticity, a proposed mechanism for motor learning and hence motor recovery [9,10], by pairing an intention to move (detected from the ongoing brain activity) with contingent somatosensory feedback from, e.g., electrical stimulation of nerves and muscles [11,12] or passive movements of the limbs through exoskeletons or rehabilitation robots [13]. BCI training has a positive effect on motor recovery after stroke (see [14] for a recent review). However, creating a stand-alone BCI training system for use in rehabilitation clinics or patients homes is difficult due to several factors. One of the factors is the setup of the EEG headset. It can be fairly time consuming to mount the EEG headset and ensure the cap is correctly placed, and a proper EEG signal quality is obtained. This is especially evident if the patients potentially should mount the EEG headset themselves [15]. These points are related to the usability of the headsets, which became a research topic when new and cheaper headsets and electrode types became available [15,16,17,18]. The usability evaluation consists of three factors: effectiveness, efficiency and satisfaction [19], and the former two factors are related to another impeding factor to create a stand-alone BCI system, the need for calibrating the BCI to ensure adequate detection performance. The non-stationarity of the electrical brain signals (electroencephalography (EEG) and electrocorticography), requires that the BCI system is calibrated to ensure adequate performance, which differs depending on the control signal and application. The BCI system performance is also affected by the signal quality, and different signal processing and pattern recognition techniques are used [20,21,22]. Several studies have investigated and compared different signal processing and pattern recognition techniques, and some studies have investigated the signal quality and BCI performance of different headsets or electrode types (dry vs. wet) [16,18,23,24,25,26,27,28,29,30,31,32,33,34,35,36,37,38,39,40,41]. The focus of these studies has primarily been on BCI control signals related to communication or control, such as P300 or steady-state visual evoked potentials (SSVEP). However, BCIs for inducing neural plasticity rely on control signals associated with movement preparation, such as movement-related cortical potentials (MRCPs) or event-related desynchronization (ERD). Hence, the ability to record MRCPs and ERD is crucial for any neurorehabilitation BCI, and this ability has been less explored in commercial headsets. A single study compared the signal quality of MRCP when recorded with two different amplifiers but with the same headset [42], and a couple of studies investigated if ERD patterns could be identified using dry and wet electrodes [27,28]. Thus, there is a need for an evaluation of different headset types that potentially could be used for BCI training in neurorehabilitation clinics. Various metrics have been used in the literature to evaluate the signal quality of the headsets or electrode types. For BCI applications, reasonable measures would be BCI performance-related metrics, such as classification accuracy or information transfer rate (especially for BCI applications within communication and control of external devices) [18,23,25,26,30,31,34,37]. The signal quality may be quantified in other ways as well. In a recent study, Oliveira et al [39] proposed a number of metrics to investigate the signal quality when comparing different headsets. These metrics include data or epoch rejection rate [16,39,40,43] and signal-to-noise ratio (SNR) or noise level [16,32,39,42,44]. This is also important for BCI applications, since the performances of pattern recognition techniques are affected by various types of noise that cannot be suppressed. Other important measures are related to the signal morphology, which can be quantified from grand averages across multiple trials (both event-related and evoked potentials) in which the amplitudes of the brain potentials are extracted, or scalp topographies in which amplitudes or spectral content are extracted from multiple channels over the scalp [32,36,37,38,39]. Lastly, it is important to evaluate the test–retest reliability of the measures [39].

In this study, the aim was to explore if MRCPs can be recorded with four different headset types. Two headsets covered the motor cortex, which is the traditional position to record MRCPs. The other two headsets were placed on the forehead and around the ear, which was preferred by stroke patients in a recent usability study [15]; however, it is not known if MRCPs can be recorded from these positions. We tested whether movement intentions (MRCPs) can be classified with respect to idle activity, which is the scenario that would be used in BCI training for stroke rehabilitation. Moreover, different signal quality measures are reported, as well as the test–retest reliability over two separate days.

## 2. Materials and Methods

### 2.1. Participants

Twelve healthy participants were included in this study (28 ± 3 years old, 2 females). All participants gave their written informed consent prior to the experiments. All procedures were approved by the local ethical committee of Region North Jutland, Denmark (N-20130081).

### 2.2. EEG Headsets

**Figure 1:**
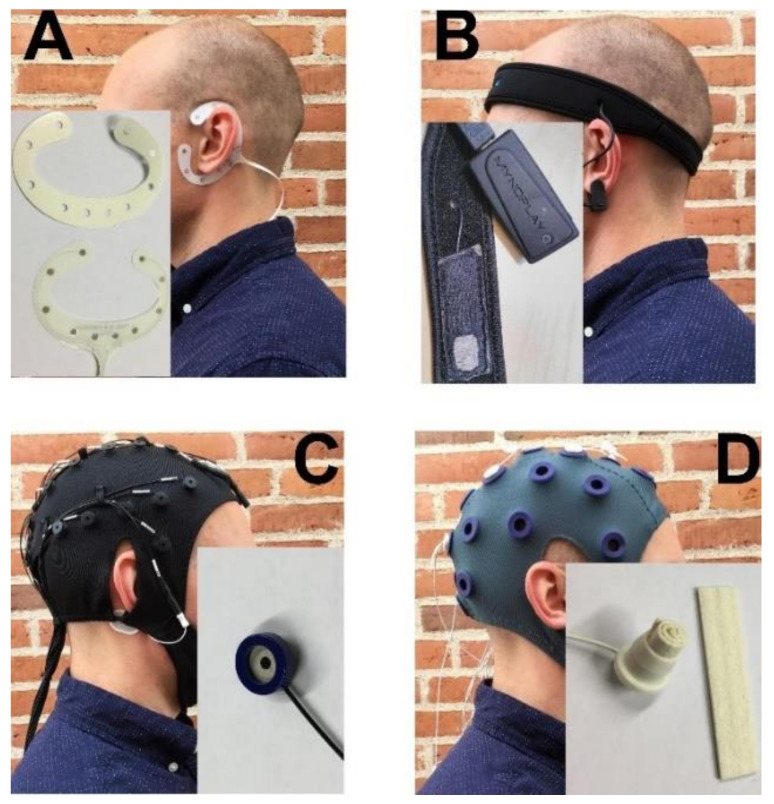
Overview of the four headsets and the electrode types. (**A**): cEEGrid from TMSi. The inset shows the sensor array and the double-sided adhesive tape. The channel in the upper part of the “C” when mounted is channel 1, and the channel numbers ascend clockwise on the electrode. (**B**): MyndBand from MyndPlay. The inset shows the dry electrode and the Bluetooth unit that transmits the data. (**C**): Quick-Cap from Compumedics. The inset shows the electrode type that is mounted in the cap. (**D**): Water-based headset from TMSi. The inset shows the electrode house and the felt insert.

Four different types of headsets were included in this study. The four headsets are shown in Figure 1.

#### 2.2.1. cEEGrid: TMSi

The cEEGrid electrode (Figure 1A) contained 10 channels surrounding the ear [45]. In this study, it was placed around the left ear (close to T9 with respect to the International 10−20 System). The electrode consisted of a flex-PCB with screen-printed silver electrodes; the electrode was attached to the user with double-sided adhesive tape, which had to be fitted accurately to the recording sites. A small amount of electrode gel (ECI Electro-Gel™) was applied to each recording site. The channels were referenced to the channel at the lower end of the “C” (channel 10) and grounded to a moist wristband (left wrist). There was no measure of electrode impedance. The signals were sampled with 2000 Hz. The signals were recorded using a Mobita® amplifier from TMSi (Tucker, GA, United States).

#### 2.2.2. MyndBand: MyndPlay

The MyndBand (Figure 1B) contained one dry electrode, which was placed on the forehead (close to F9 with respect to the International 10−20 System). The electrode was fixed with a neoprene headband. The signals were referenced to the left earlobe with an ear clip; there was no information about a ground electrode. A tool for measuring the impedance was available in the recording software; the impedance could be in the following range: very poor–perfect. The impedance was perfect for all participants. However, it is not known what “perfect” corresponds to in terms of kΩ. The signals were sampled at 512 Hz.

#### 2.2.3. Quick-Cap: Compumedics

The Quick-Cap (Figure 1C) electrodes covered the following positions with respect to the International 10–20 System: F3, Fz, F4, C3, Cz, C4, P3, Pz, and P4. The channels were referenced to Pz and grounded at AFz. The electrodes were filled with conductive gel (ECI Electro-Gel™) to establish contact between the electrodes and the scalp. A tool was available in the recording software to measure the impedance; the impedance of all channels was below 5 kΩ in all experimental sessions. The signals were sampled at 500 Hz. The signals were recorded using a Nuamp amplifier (EEG amplifiers, Nuamps Express, Neuroscan).

#### 2.2.4. Water-Based Electrodes: TMSi

The water-based electrodes (Figure 1D) were placed in the following positions with respect to the International 10–20 System: F3, Fz, F4, C3, Cz, C4, P3, Pz, and P4. They were referenced to Pz, and they were grounded to a moist wristband (left wrist). The electrode consisted of a felt insert that had to be placed in an electrode house and soaked in water before placing it in the cap. There was no measure of electrode impedance. The signals were sampled at 2000 Hz. The signals were recorded using a Mobita® amplifier from TMSi.

### 2.3. Experimental Procedure

The participants participated in an experiment consisting of two experimental sessions, which were separated by at least 24 h. They sat in a comfortable chair and performed 100 cued ballistic palmar grasps of the right hand when continuous EEG was recorded. The hand was opened immediately after the grasp was performed; i.e, the contraction was not maintained. The participants were instructed to perform the movements as rapidly as possible and relax immediately after the grasp was performed. This movement type was performed for each of the four headsets, so, in total, 4 × 100 movements were performed in each of the two experiments. The order of the headsets was randomized on both recording days using Random.org. The participants were given two cues; one cue three seconds prior to the second cue, which indicated the onset of the task. The participants were instructed to sit as still as possible and avoid any eye movements and contractions of facial muscles. A trigger was used to mark the continuous EEG at the first cue; this was used to divide the continuous EEG into epochs. Each movement was separated by ten seconds. It took ~17 min to perform 100 movements. There was a break between the tests of the different headsets in which the subjects washed their hair.

### 2.4. Data Analysis

The EEG signals were analyzed in two different ways; 1) an analysis of the signal morphology, and 2) discrimination between movement intention and idle activity. For the signal quality analyses, the signals from Cz, or channel two for the cEEGrid, were used, since the pre-movement components of the MRCP can be recorded over the midline regardless of the site of movement [46]. For the classification of movement intentions and idle activity, all available channels were used. All analyses were performed in MATLAB 2019B (MathWorks).

#### 2.4.1. Pre-Processing

**Figure 2:**
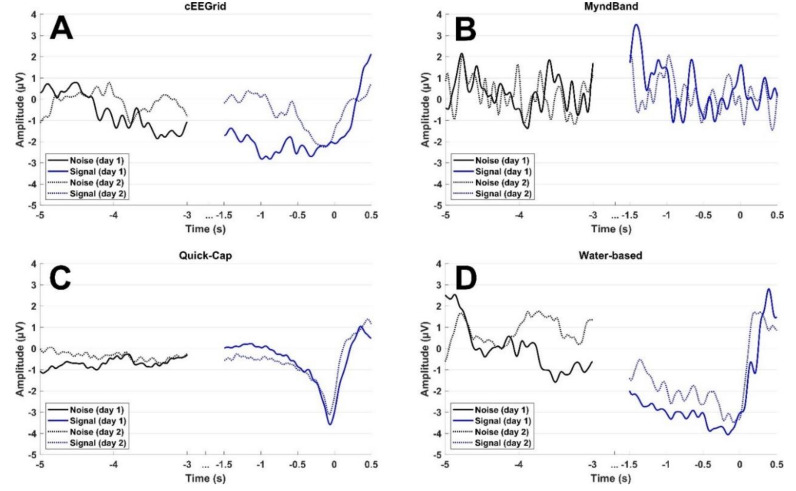
Grand average across participants for each headset and each day. “0 s” is the task onset. For the Quick-Cap and water-based electrodes, Cz was used, whereas channel 2 was used for the cEEGrid and the single electrode on the forehead for the MyndBand.

The signals were band-pass-filtered from 0.05−10 Hz using a fourth-order zero-phaseshift Butterworth filter and downsampled to 500 Hz. The epochs were divided into “idle/noise” and “signal” epochs. Idle epochs were extracted from −5 to −3 seconds prior the movement onset, whereas the signal epochs were extracted from −1.5 to 0.5 s with respect to the task onset (0 s was the task onset). See Figure 2.

#### 2.4.2. Signal-to-Noise Ratio, Epoch Rejection, and Peak Amplitudes

Initially, the signal and idle epochs that exceeded ±150 µV in peak–peak amplitude were rejected in the Cz channel (Quick-Cap and Water-based electrodes), channel 2 (cEEGrid, close to T9 according to the International 10−20 System) and in the single MyndBand channel (close to F9 according to the International 10−20 System). The analyses in this subsection were based on the specified individual channel for each headset. If more than 80% of the epochs were rejected, all data from that participant was removed from further analysis (see Table 1). The average of the signal and average of the idle epochs were calculated, and the root-mean-square (RMS) value was calculated of the two averages. The ratio between the signal and idle/noise RMS values were used as an estimate of the SNR. The average across the signal epochs was computed and the average amplitude was calculated from −0.2 to 0.2 s around the movement onset. The number of rejected epochs, SNR and averaged peak amplitudes were used as measures of signal quality.

#### 2.4.3. Feature Extraction and Classification

Initially, the signal and noise epochs that exceeded ±150 µV in peak–peak amplitude in any of the recorded channels were excluded from further analysis. The feature extraction was performed for each channel. The feature extraction was based on previous studies, where MRCPs and ERD were detected from time domain analysis [47], frequency domain analysis [48], and template matching [49]. In the time domain, the mean amplitudes were extracted for 0.5-second windows without overlap and used as features, and the difference between the average amplitude in the first half and second half of the epoch. The power spectral density was estimated for the entire epoch with a 1-second Hamming window with 0.5-second overlap; the features were the power spectrum in 1-Hz bins from 6 to 30 Hz. For the template matching, the epochs were filtered from 0.05−10 Hz. An average of the signal epochs in each channel was obtained, and the cross-correlation was calculated between the template and the epochs at zero time lag. The classification was performed using a Random Forest classifier in a leave-one-out cross-validation scheme. The classifier was trained using 512 trees. The classification accuracy and number of rejected epochs are reported.

### 2.5. Test–Retest Reliability

The test–retest reliability was estimated using the intraclass correlation coefficient between the two recording days for participants that had a complete dataset using a 2-way mixed effect model with absolute agreement (IBM®, SPSS®). The intraclass correlation coefficient was calculated for SNR, average peak amplitudes and classification accuracies.

### 2.6. Statistics

Five two-way repeated measures analyses of variance (ANOVA) were performed with ‘Headset’ (four levels: cEEGrid, MyndBand, Quick-Cap, and Water-based) and ‘Time’ (two levels: Day 1, and Day 2) as factors on the following measures: 1) SNR, 2) amplitude, 3) number of rejected epochs (single-channel), 4) classification accuracy, and 5) number of rejected epochs (multiple channels). If the assumption of sphericity was violated, the Greenhouse–Geisser correction was applied. Significant tests were followed up with a posthoc test with Bonferroni correction. Significant test statistics were assumed when *p* < 0.05. The effect size was reported as well, using the partial eta squared value (η2). 

## 3. Results

**Figure 3:**
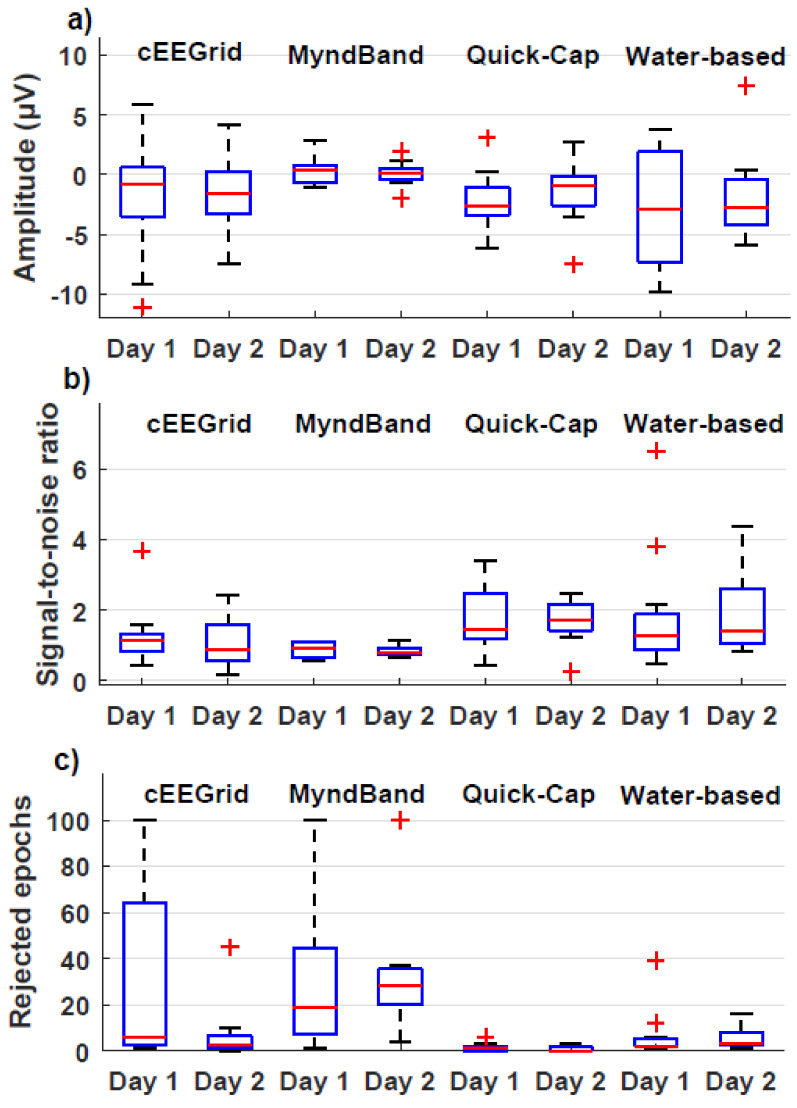
Boxplots with quartiles for the signal quality analyses. (**a**): Average amplitude around the movement onset; (**b**): Signal-to-noise ratio; (**c**): Number of rejected epochs for a single channel.

**Figure 4:**
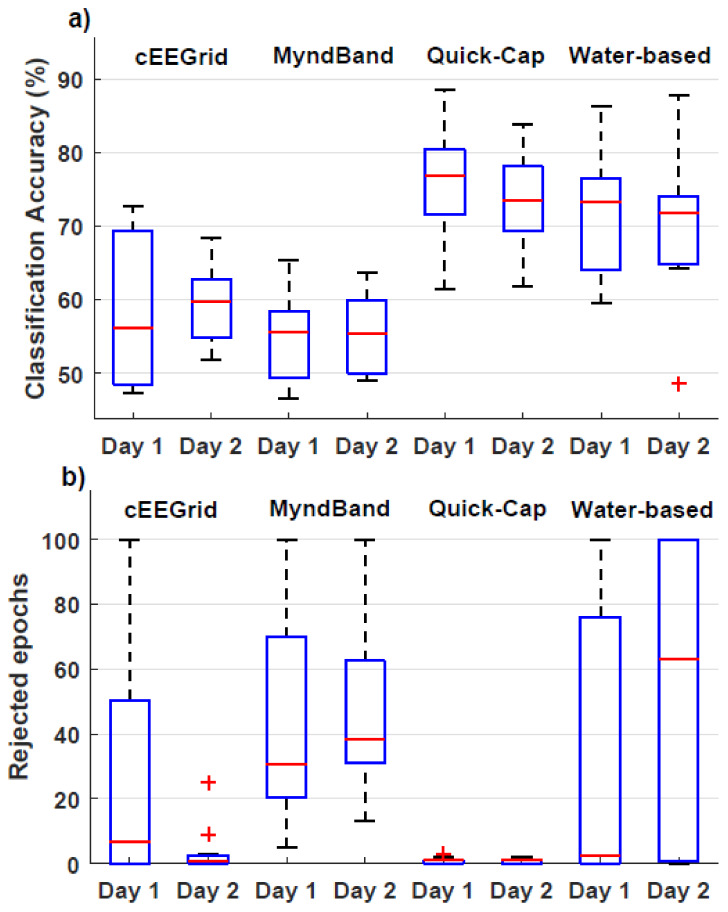
Boxplots with quartiles for the classification accuracies (**a**) and number of rejected epochs (**b**).

The results are summarized in Figure 3 and Figure 4 and Table 1, Table 2 and Table 3.

### 3.1. Signal Quality

The results of the signal quality analyses are presented in Table 1 and Figure 2 and Figure 3. The average amplitude around the movement onset was most prominent for the Quick-Cap and Water-based headset. It was expected to see a negative potential, such as the one shown in Figure 2C. The peak amplitudes recorded from the other two headsets were less prominent, and the clear MRCP morphology was absent. There was no interaction between headset and time (F_(3,21)_ = 0.51; *p* = 0.68; η^2^ = 0.07), and no effect of time (F_(1,7)_ = 0.38; *p* = 0.56; η^2^ = 0.05) and headset (F_(1.7,12.2)_ = 1.95; *p* = 0.19; η^2^ = 0.22).

The SNR was highest for the Quick-Cap and the water-based headset, whereas the other two had similar SNRs. The statistical analysis showed no interaction between headset and time (F_(1.2,7.1)_ = 0.03; *p* = 0.89; η^2^ = 0.06) and no effect of time (F_(1,6)_ = 0.01; *p* = 0.91; η^2^ = 0.02), but the effect of headset was significant (F_(3,18)_ = 6.67; *p* = 0.003; η^2^ = 0.53). The posthoc test revealed that the SNR associated with Quick-Cap was higher than the SNR obtained with the MyndBand.

The median number of rejected epochs was in the range of 0−6 rejected epochs for the Quick-Cap, water-based headset, and cEEGrid, whereas the median number of rejected epochs for the MyndBand was 19 and 28. This is also reflected in the number of participants that were rejected based on the criterion of 80% of the samples that should be within ±150 µV peak–peak amplitudes. There was no interaction between headset and time (F_(1.3,14.2)_ = 1.38; *p* = 0.27; η^2^ = 0.11) and no effect of time (F_(1,11)_ = 4.37; *p* = 0.06; η^2^ = 0.28), but the effect of headset (F_(1.9,20.6)_ = 11.71; *p* < 0.001; η^2^ = 0.52) was significant. The posthoc test showed that more epochs were rejected for the MyndBand compared to the Quick-Cap and water-based headset, and more epochs were rejected for the water-based headset than the Quick-Cap.

### 3.2. Movement Intention vs. Idle Classification

The results are presented in Table 2 and Figure 4. The number of rejected epochs was high for the MyndBand, and the 75% quartiles for the water-based and cEEGrid headset. This is also reflected in the number of participants that were excluded from further analysis (more than 80% of the epochs were rejected). The accuracies were close to the significance threshold of random classification (chance level = 50%), calculated with 95% confidence limits (threshold for signficance = 60%) [50] for the cEEGrid and MyndBand, whereas accuracy was well above the threshold for significance for the Quick-Cap and Water-based headset. They had similar median classification accuracies, 74%−77% and 72%−73% for the Quick–Cap and water-based headset, respectively, on the two different days, but the classification accuracies were based on fewer samples and subjects for the water-based headset. The statistical analyses revealed no interaction between headset and time (F_(3,9)_ = 0.25; *p* = 0.86; η^2^ = 0.08) and no effect of time (F_(1,3)_ = 0.09; *p* = 0.79; η^2^ = 0.03) and headset (F_(3,9)_ = 3.41; *p* = 0.07; η^2^ = 0.53). However, the sample size was limited. For the number of rejected epochs, there was no interaction between headset and time (F_(2.1,23.4)_ = 2.36; *p* = 0.09; η^2^ = 0.18), and no effect of time (F_(1,11)_ = 0.06; *p* = 0.81; η^2^ = 0.006), but the effect of headset was significant (F_(3,33)_ = 10.25; *p* < 0.001; η^2^ = 0.48). The posthoc analysis showed that more epochs were rejected for the water-based headset and Myndband compared to the Quick-Cap, and that more epochs were rejected for the MyndBand compared to the cEEGrid.

### 3.3. Test–Retest Reliability

The test–rerest reliability was assessed using the intraclass correlation coefficient, and the results are presented in Table 3. A moderate (intraclass correlation coefficient: 0.50−0.75) to good (intraclass correlation coefficient: 0.75−0.90) reliability was obtained for the Quick-Cap, whereas poor (intraclass correlation coefficient <0.50) to moderate reliability was obtained for the other headsets [51]. The negative intraclass correlation coefficients are likely due to bad estimates from a limited sample [52], where the mean-square error is larger than the mean square of the rows [51]. The negative values are obtained for the headsets where subjects were excluded (see Table 2).

## 4. Discussion

In this study, the aim was to test four different types of headsets and electrodes. The results of the evaluation suggest that it is important to have electrodes that cover the motor cortex area [46], and that gel-based electrodes are superior to the alternatives, in order to discriminate between movement intentions of the hand and idle activity (median classification accuracy of 77% and 74% on day 1 and 2, respectively). The MRCP morphology was most cleary seen for the headset that covered the motor cortex and used conductive gel. It must be stated that some of the headsets were not developed for recording movement-related activity, which was also reflected in the results. However, it was important to include these headsets, since stroke patients in a comparative study preferred them [15]. 

The classification between movement-related and idle activity was not significantly higher than chance level (when calculated with 95% confidence limits [50]) for the MyndBand and cEEGrid. This is likely due to the electrode positions, which were too far away from the motor cortex to register the MRCP. The water-based headset covered the motor cortex, and the classification accuracies were significantly higher than chance level (threshold for signficance = 60% [50]). However, many epochs were removed from the analysis due to large amplitudes in the EEG, which potentially arose from electrodes losing contact with the skin. Further processing can be done to remove bad channels and perform the classification based on a single or reduced number of channels. The most reliable classification of movement-related activity was obtained using the Quick-Cap with gel-based electrodes. The median classification accuracy was 77% and 74% for day 1 and day 2, respectively. These accuracies are similar to what has been reported previously [47,48,53]. From a BCI training point of view, the classification accuracy/BCI system perfomance needed for inducing plasticity is not known [7], but the accuracies obtained for the water-based electrodes and Quick-Cap are higher or similar to the BCI system performance that has been reported to induce neural plasticity in previous studies (true positive range: 67%−85%) [11,12,13]. It has previously been indicated that the BCI system performance and induction of plasticity are positively correlated [11]; therefore, there is an incentive for further improving the classification accuracies and hence the BCI system performance. One way to do this is by applying a spatial filter [20,54]; however, this can only be done when multiple channels are recorded. Besides neurorehabilitation, these results are also relevant for communication or internet browsing for late-stage ALS patients that will be able to produce similar slow cortical potentials. In a simulation study [55], it was shown that an accuracy of 75% can be used to browse the internet, using slowly developing control signals such as the MRCP or ERD. However, to increase the speed of browsing or communication (i.e. increase the information transfer rate) evoked potentials such as P300 should be used if the user is able to operate them [56]. 

### Limitations and Future Perspectives

A limiting factor that could affect the number of rejected epochs in the study was the choice of amplitude threshold. The threshold of 150 µV could be too high to exceed for the cEEGrid electrode, which would affect the classification analysis, since the accepted epochs would still contain noise. This could also account for the high number of rejected epochs for the water-based electrodes and the MyndBand. Another approach could be to use a data-driven threshold using X times the standard deviation; X could be in the range of, e.g., 3–5, depending on how conservative the noise rejection should be. Only the headset with electrodes covering the motor cortex region and with conductive gel applied had clear MRCP waveforms, but both headsets that covered the motor cortex region had significant decoding accuracies; this suggests that it is necessary to use headsets with motor cortex region electrodes in order to decode MRCPs. If gel-based electrodes are used, patients may need to wash their hair after using the BCI, unless a limited number of channels are used. It has previously been shown that a single channel is sufficient to detect the MRCP [53]. In the current study, it was an experimenter with several years of experience within EEG recordings and BCI research who mounted the headsets. If BCI training is going to be performed in rehabilitation clinics and the patient’s home then it would be important to test how much time it will take for rehabilitation professionals, caretakers, or relatives to learn to setup the headset correctly and obtain signals of sufficient quality to be classified correctly. In this study, different headsets and electrode types were chosen to test if movement-related activity could be recorded and classified, although they were not designed for that specific purpose. There exist other headsets (and new ones are emerging) that are candidates to record movement-related activity, and, therefore, it would be relevant to perform further comparative studies between headsets to identify the optimal headset in terms of signal quality, comfort, setup difficulty and price, to increase the likelihood of potential end-users adopting the BCI technology. 

## 5. Conclusions

It is concluded that it is necessary to record signals from the motor cortex area to be able to detect movement intentions. The results indicate that the most reliable classification accuracies are obtained with gel-based electrodes. These results may have implications for the choice of headset for BCI applications within neurorehabilitation, or applications that require an estimate of MRCPs.

## Figures and Tables

**Table 1:** Results of the signal quality analyses for the four headsets. The signal-to-noise ratio (SNR), average amplitude around the movement onset, number of rejected epochs, and number of rejected participants are presented for both experimental sessions. The results are presented based on Cz (water-based electrodes and Quick-Cap) or the channel closest to that (MyndBand and cEEGrid).

	SNR		Amplitude (µV)		# Rejected Epochs		# Excluded Participants	
	Day 1	Day 2	Day 1	Day 2	Day 1	Day 2	Day 1	Day 2
cEEGrid	0.8/1.2/1.4	0.6/0.9/1.6	−4.9/−0.8/0.9	−3.6/−1.6/0.6	2/6/65	1/3/7	2	0
MyndBand	0.6/0.9/1.1	0.7/0.8/0.9	−0.7/0.3/0.9	−0.6/0.2/0.6	7/19/45	18/28/36	2	1
Quick-Cap	1.1/1.5/2.5	1.4/1.7/2.2	−3.4/−2.6/−0.9	−2.6/−1.0/0.0	0/1/2	0/0/2	0	0
Water-based	0.8/1.3/2.0	1.0/1.4/2.7	−7.4/−2.9/1.9	−4.3/−2.7/−0.3	2/2/6	2/3/9	0	0
	25% / Median / 75%							

**Table 2:** Results of the classification analyses for the four headsets. The classification accuracy and number of rejected epochs are presented for both experimental sessions. The results are based on all available channels; therefore, the number of rejected epochs differ from Table 1, which was based on a single channel.

	Classification Accuracy (%)		# Rejected Epochs		# Excluded Participants	
	Day 1	Day 2	Day 1	Day 2	Day 1	Day 2
cEEGrid	48/56/70	55/60/63	0/7/54	0/1/3	2	0
MyndBand	49/56/59	50/56/60	19/31/85	31/39/70	3	2
Quick-Cap	70/77/82	69/74/78	0/1/1	0/1/1	0	0
Water-based	64/73/78	65/72/75	0/3/88	0/63/100	3	4
	25% / Median / 75%					

**Table 3:** Test–retest reliability of the signal-to-noise ratio (SNR), average amplitude around the movement onset and the classification accuracies.

Intraclass Correlation Coefficient (ICC)			
	ICC_SNR	ICC_Amplitude	ICC_Classification Accuracy
cEEGrid	−0.3	0.32	0.63
MyndBand	0.43	−0.21	0.33
Quick-Cap	0.78	0.83	0.59
Water-based	−0.29	0.06	−0.11
